# The Impact of WHO-2023 Malnutrition Criteria on Caseload of Infants Aged Under Six Months: Secondary Data Analysis

**DOI:** 10.3390/children12020118

**Published:** 2025-01-22

**Authors:** Ayenew Negesse, Tsinuel Girma, Beruk Berhanu Desalegn, Melkamu Berhane, Marko Kerac

**Affiliations:** 1 Academic Center of Excellence for Human Nutrition, School of Nutrition, Food Science and Technology (SNFST), Hawassa University, Hawassa P.O. Box 05, Ethiopia; beruk@hu.edu.et; 2 Department of Human Nutrition, College of Medicine and Health Science, Debre Markos University, Debre Markos P.O. Box 269, Ethiopia; 3 Department of Paediatrics and Child Health, Faculty of Medical Science, Jimma University, Jimma P.O. Box 378, Ethiopia; tgirma@hsph.harvard.edu (T.G.); melkamu.berhane@ju.edu.et (M.B.); 4 Harvard Chan School of Public Health, Addis Ababa P.O. Box 1242/5646, Ethiopia; 5 London School of Hygiene and Tropical Medicine, Keppel Street, London WC1E 7HT, UK; marko.kerac@lshtm.ac.uk

**Keywords:** malnutrition, caseload, anthropometric indicators, infants u6m Venn diagrams

## Abstract

Background/Objectives: The 2023 World Health Organization (WHO) guideline on infants aged under six months (u6m) wasting now includes mid-upper arm circumference (MUAC) and weight for age Z score (WAZ) as malnutrition treatment programme enrolment criteria. Evidence for the new criteria and optimal cut-offs is, however, sparse. We aimed to explore the potential caseload implications of the new criteria and explore how different cut-offs might affect them. Methods: We conducted a secondary analysis of data from 1048 Ethiopian infants u6m using STATA software version 17. Frequency tables and percentages were used to present malnutrition across various characteristics. The chi-square test with 95% confidence intervals (CIs) at a *p* value of <0.05 was used to compare infant caseload identified by the WHO-2023 versus WHO-2013 criteria. Results: We found substantial overlaps among anthropometric indicators of malnutrition in infants defined by the WHO-2023 programme enrolment criteria. New WHO criteria result in a larger potential caseload (19.2% for 6 weeks to 6 months and 15.1% for infants under 6 weeks) compared with WHO-2013 criteria (2.4%). Whilst there are marked overlaps between low WAZ, low WLZ, and low MUAC, they capture different infants. An MUAC of <110 mm alone would capture only a third of all cases identified by WAZ and/or WLZ < −2. Conclusions: In Ethiopia, the WHO-2023 criteria markedly increase malnutrition caseloads compared with WHO-2013 criteria. There might be a case for increasing MUAC thresholds in MUAC-focused programs where WLZ or WAZ measurements are difficult. Future longitudinal data are needed to know which criteria best identify infants at highest risk of mortality/morbidity/poor development.

## 1. Introduction

The first six months of age is the fastest growth period in human life [1]. It is a critical period of growth and development with a high risk of mortality and morbidity, including long-term morbidity if nutrition is suboptimal [2,3,4]. Malnutrition is thus an important issue in this age group, and yet for many years, it has often been neglected in global policy and practice [5,6]. This occurs despite the problem being common.

A recent analysis of datasets from 54 low- and middle-income countries found that several forms of malnutrition were common among infants under six months (u6m) of age: 20.1% were underweight, 21.3% were wasted, and 17.6% were stunted [7]. Other recent evidence from India also showed that 36.8% of infants u6m were underweight and 20.2% were wasted [8]. Both findings show that malnutrition in infants u6m still continues to be a major global health problem.

The old 2013 World Health Organization (WHO) guideline for the management of infant and child malnutrition [9] focused on low weight-for-length Z score (WLZ) for enrolling infants u6m into treatment programmes. Recently, there has been growing evidence that other indicators, notably low weight-for-age Z score (WAZ) and low mid-upper arm circumference (MUAC), identify at-risk infants u6m far better than WLZ [10,11].

The new WHO-2023 guideline on child malnutrition expands the enrolment criteria for infants u6m, terming them as infants “at risk of poor growth and development” [12]. Alongside some other criteria (infants with poor growth based on sequential measures; infants with disability and other clinical/maternal risk factors for poor growth and development; and infants at risk due to poor birth outcomes), new anthropometric criteria based on a single measure now include:

– Weight-for-age Z score (WAZ) < −2 SD (underweight infants); or
– Weight-for-length Z score (WLZ) < −2 SD (wasted infants—an expansion from the old 2013 criteria, which only focused on severely wasted infants, WLZ < −3); or
– Nutritional oedema; or
– Mid-upper arm circumference (MUAC) < 110 mm for infants between 6 weeks to less than 6 months of age (there is no MUAC recommendation for younger infants).

MUAC is of particular interest to many programmers as it is quick, easy, and reliable to assess, and in addition, it works well for identifying high-risk children [13,14,15]. The WHO-2023 recommendation for MUAC threshold for infants u6m is, however, based on very limited evidence. Indeed, the optimal thresholds for all the new indicators are not set by a direct examination of optimal sensitivity/specificity for identifying key outcomes like mortality, nor are the caseload implications known.

For instance, while there are currently no specific reports of malnutrition caseloads as per the new WHO-2023 criteria or reports of the extent of anthropometric overlaps in capturing cases [12], experts in the field are actively discussing the necessity of tailoring this global guideline to suit the unique contexts of different countries for their effective implementation and impact [16,17].

Additionally, member states and implementing partners around the world, particularly in low-resource settings like Ethiopia, may confront problems in scaling up existing programs through the empirically set new WHO-2023 infant malnutrition caseload assessment criteria [12,16,17,18].

These issues present major challenges for policymakers and program managers in adopting the new guideline. Another key question relates to the fact that malnutrition treatment programmes for older children often use MUAC as a sole entry/exit criterion. Future programmes for infants would likely seek to operate the same way. Policymakers and programme managers thus need to know how low MUAC, at different thresholds, overlaps with other indicators.

These considerations highlight the need for policy and practice-relevant research to better understand malnutrition prevalence in infants u6m and program caseload implications that would occur if infants are identified as per the new WHO-2023 anthropometric criteria, including specific focus on different MUAC thresholds [12]. This is vital for building effective, evidence-based interventions that could be widely implemented in Ethiopia and other similar countries [16,19], thereby contributing to the Sustainable Development Goal nutrition target of “Zero Hunger” by 2030 [20].

In response to the above evidence gaps, we aimed to investigate how the criteria for infants u6m in the new WHO guideline might affect the treatment program caseloads of infants u6m at risk of poor growth and development. The specific objectives are to:

(1) Determine the magnitude and compare potential caseload implications of malnutrition among infants u6m using the new WHO-2023 criteria; and
(2) Explore the overlap between different MUAC thresholds and low WLZ and low WAZ.

We anticipate that this original study will help national programmers and policymakers around the world, specifically for low-resource settings like Ethiopia, as they adopt and rollout the new WHO-2023 guideline to better support the effective management of infants u6m at risk for mortality/morbidity/development.

## 2. Materials and Methods

### 2.1. Study Design, Areas, and Period

This is a secondary analysis of previously reported cross-sectional data collected from health centres in Ethiopia [21]. We build upon our previous work by exploring different MUAC thresholds in much more detail and by focusing on new WHO criteria.

Our data were from two areas of Ethiopia. One area was Jimma, representing relatively livelihood-secure communities in the central highlands of Southwest Ethiopia [22,23,24,25,26,27,28,29]. The other was Deder, a more livelihood-insecure area in the lowlands of Eastern Ethiopia [30,31,32,33,34]. Data were collected between 12 October 2020 and 29 January 2021.

### 2.2. Population, Sample Size, and Sampling Procedure

**Figure 1:**
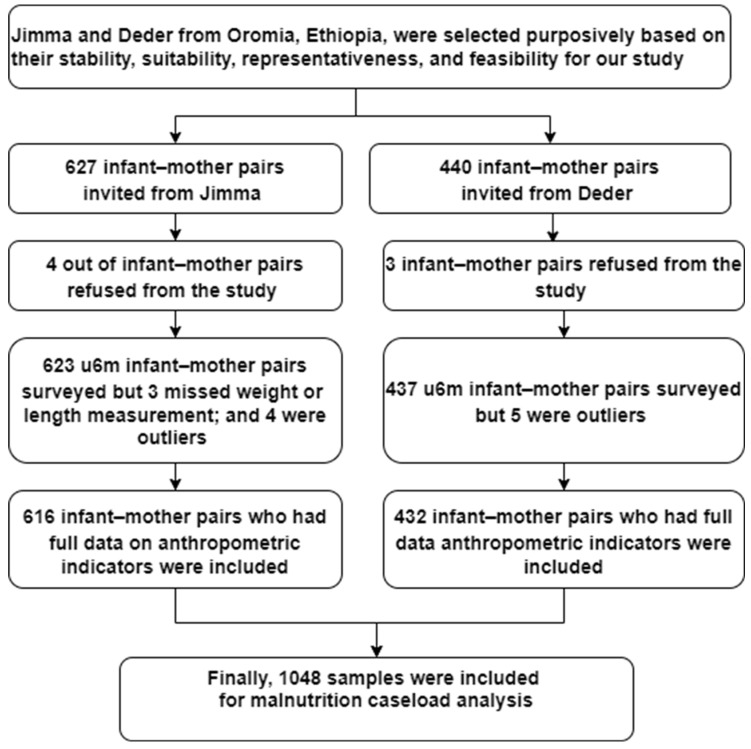
Schematic presentation of clinic-level patient sampling procedure.

Figure 1 summarises the clinic-level patient sampling procedure and total samples included in our malnutrition caseload analysis. Our data were from 1048 infant–mother pairs who visited Jimma and Deder health centres for delivery, immunisation, growth monitoring, and acute disease treatment. Full details regarding study selection, sample size, sampling technique, and inclusion criteria are found in our original paper [21]. In brief, as this study focuses on malnutrition caseload at health centres, specifically how the new WHO-2023 criteria impact at risk infant for poor growth and/or development eligible for treatment programs, we used data from our original study, which was also focused on the issue of malnutrition in infants u6m [21]. The sample size estimation technique and sampling procedures are summarised as follows:

Due to the lack of previous data on infants u6m with malnutrition attending health centres, we assumed 50% prevalence and 3% precision to estimate a sufficiently robust sample [35]. This translated to a total of 1067 infants, with an average of 60 per health centre. For logistical reasons, we assumed an average weekly visit of 30 infants at each facility and established a two-week data collection period.

We included all eight health centres in Deder. In Jimma, we assessed the registers of all 124 health centres to gather eligibility information regarding their caseload and accessibility. We excluded 60 health centres due to inadequate caseloads and an additional 7 health centres for their inaccessibility. Finally, we ranked the remaining 57 health centres by caseload, and we randomly selected 10 out of the top 50% to ensure a representative sample for our study.

### 2.3. Data Collection, Measurement, and Quality Management

Details of the original data collection procedures are described in full in our original report [21]. In summary, we trained twelve nurses as data collectors and had 4 supervisors with Masters degree in any health science to ensure consistent and high-quality data collection. The training included anthropometric measurements (weight, length, and MUAC), infant feeding practices, economic and demographic data, use of digital data gathering devices, informed consent, and clinical history of infant–mother pairs.

Those trained data collectors collected the data through local Amharic and/or Afaan Oromo languages via the tablet-based ‘Research Electronic Data Capture’ (REDCap) project system (https://redcap.am.lshtm.ac.uk/redcap/ accessed on 14 May 2021). Questions were asked of the infants’ carers and included household/demographic data; data about the mother/main carer; and clinical, nutritional, and anthropometric data about the infants. The infant was undressed, and their weight was measured to the nearest 0.005 kg if weighing < 10 kg, or to the nearest 0.01 kg if weighing ≥ 10 kg, using a digital weigh scale (Seca 354), which is precise and easy to use in paediatric clinical settings.

We measured length to the nearest 0.1 cm using a UNICEF length/height board. MUAC (mid-upper arm circumference) was also measured to the nearest 0.1 cm using a UNICEF-supplied measurement tape. Anthropometric measurements were taken in pairs, with remeasurement being performed if it was outside the set limits of agreement as per WHO child growth standards protocols [36].

Based on the 2006 WHO Child Growth Standards [36], the weight, length, and age data were used to calculate the anthropometric indicators WAZ and WLZ score, respectively, by using the zanthro command package in STATA [37]. Underweight was defined as having a WAZ score of <−2 and wasting as a WLZ score of <−2. With these, an assessment of potential caseload as per WHO-2023 anthropometric definitions was performed. Withering is similar to wasting in that it describes the physical deterioration or loss of muscle mass and fat but is assessed by low MUAC rather than low WLZ [12]. The WHO-2023 guideline set an MUAC threshold of <110 mm to define withering in infants 6 weeks to 6 months and is used alongside low WAZ and WLZ scores to define infants at risk of poor growth and development who are eligible for treatment programme enrolment and support [12].

### 2.4. Statistical Analysis

Statistical analysis was performed using STATA software version 17 (STATA 17, College Station, TX, USA). As per WHO-2023 malnutrition diagnostic criteria for infants u6m, we have first separated the data as: those < 6 weeks and >= 6 weeks to 6 months. We calculated Z scores for the required anthropometric indicators using STATA’s zanthro statistical program. Then, we estimated different forms of malnutrition such as wasting (WLZ < −2), underweight (WAZ < −2), and ‘withering’, defined as MUAC < 110 mm in infants ≥ 6 weeks to < 6m. Proportional Venn diagrams generated by the pvenn and venndiag STATA sub-commands were used to visually illustrate the overlap of those anthropometric indicators for diagnosing malnutrition caseload in infants u6m with different age groups and across the different MUAC thresholds.

Frequency tables and percentages were employed to present the degree of malnutrition across the different variables. Plus, to assess significant differences between malnutrition caseload identified by the new WHO-2023 criteria versus the old WHO-2013 criteria, we considered the chi-square test with the 95% confidence intervals (CIs) at a *p* value threshold of <0.05.

To obtain an overall infant u6m malnutrition caseload estimate, we used recent birth rate data of Ethiopia reported by the United Nations Population Fund (UNPFA) in 2023 [38]. Total numbers of infants u6m were calculated as total annual births divided by 2.

## 3. Results

### 3.1. Socio-Demographic and Economic Characteristics of Infant–Mother Pairs

Table 1 summarises the socio-demographic and economic characteristics of u6m infant–mother pairs. Of the participants in the survey, 437 (41.2%) were from Deder health centres, whereas 623 (58.8%) were from Jimma ones. Almost half of infants, 454 (42.8%), were aged between 2 and 4 months.

Over half, 588 (55.5%), of infants were males. Most mothers, 966 (91.1%), were over 19 years old, and 862 (81.3%), married for the first time before the age of 19 [Table 1]. Most mothers, 941 (89.4%), also reported normal scores on our PHQ-9 mental health questionnaire, while 485 (45.8%) of mothers and 452 (45.4%) of household heads had completed primary school education. Nearly half of infants, 519 (49%), were exclusively breastfed, and 611 (57.6%) of households were using non-tap water for household consumption [Table 1].

### 3.2. Infant Malnutrition Caseloads at MUAC < 110 mm Against WLZ (−2) and WAZ (−2) WHO-2023 Criteria

**Figure 2:**
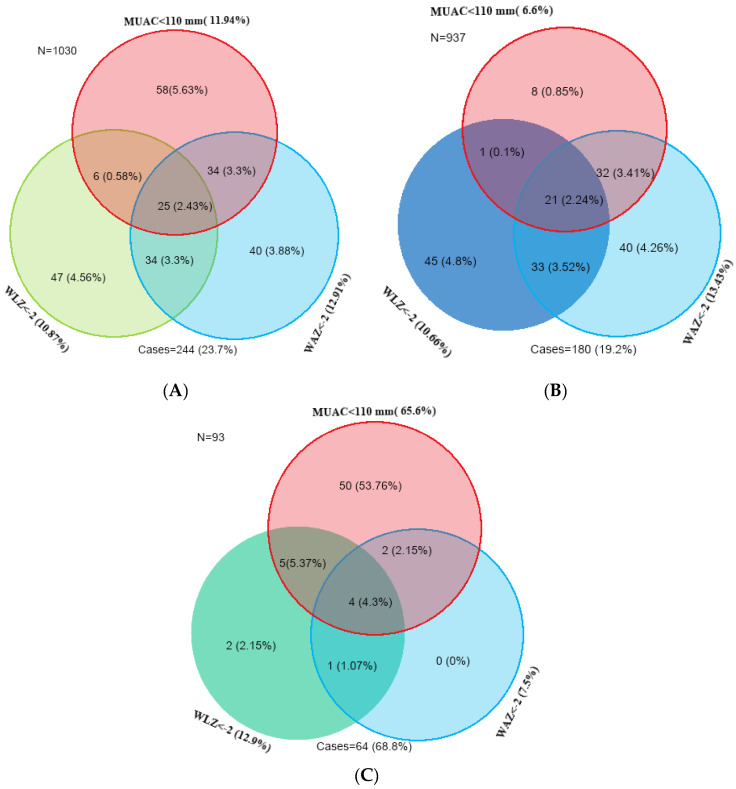
Overlap of malnutrition caseload across the different anthropometric indicators in infants u6m (all infants, those aged between 6 weeks to 6 months and younger infants aged < 6 weeks) defined by the new WHO-2023 criteria. (**A**) Overlap of anthropometric deficit for all infants (0–6 months) (MUAC < 110 mm, WAZ < −2, and WLZ < −2). (**B**) Overlap of anthropometric deficit for >= 6 weeks to 6 months infants (MUAC < 110 mm, WAZ < −2, and WLZ < −2). (**C**) Overlap of anthropometric deficit for < 6 weeks infants (MUAC < 110 mm, WAZ < −2, and WLZ < −2).

Figure 2 shows the overlap of anthropometric deficit across the different anthropometric indicators among infants u6m in resource-limited settings against the new WHO-2023 SAM management guideline. In all infants aged 0–6 months, we found that 11.9% of infants were classified as withering (MUAC < 110mm), 12.9% were underweight (WAZ < −2), and 10.9% were wasted (WLZ < −2) [Figure 2A].

We also performed a separate analysis based on the infant’s age, applying the same MUAC cut-off along with WLZ and WAZ anthropometric indicators for ‘infants at risk of poor growth and development’. For infants aged 6 weeks to 6 months, only 6.6% of infants u6m had an MUAC of <110 mm; 10.7% had a low WLZ; and 13.4% a low WAZ. Thus, 19.2% overall would have been eligible for care as per the ‘infants at risk of poor growth and development’ case definition [Figure 2B].

Employing the same MUAC threshold of <110 mm for infants aged < 6 weeks identified, 65.6% of infants u6m would be flagged vs. only 12.9% using low WLZ and 7.5% with low WAZ. Thus, 68.8% of infants aged < 6 weeks could be eligible for care, although the WHO does not currently recommend using MUAC at all in this youngest age group [Figure 2C].

### 3.3. Change in Proportion of Malnutrition Overlap of Infants u6m Against the Low WAZ and WLZ (−2, −3) Standards

**Figure 3:**
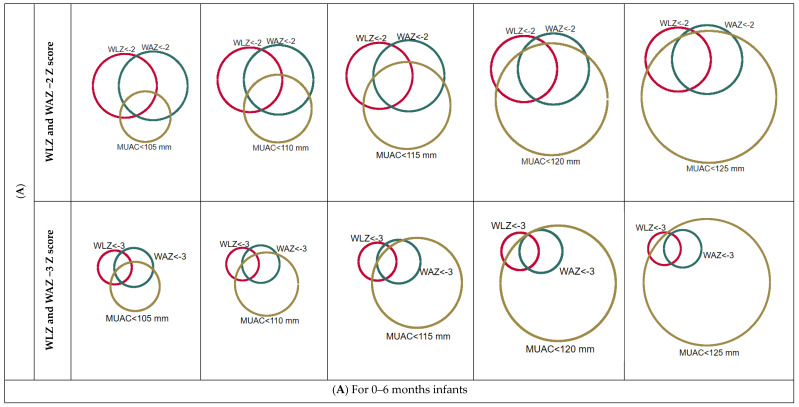

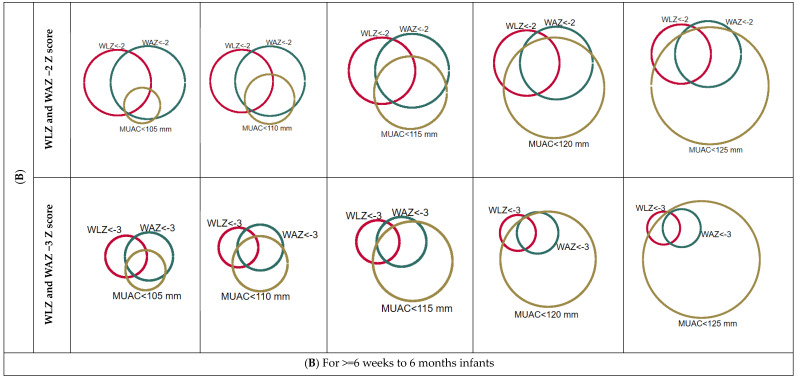

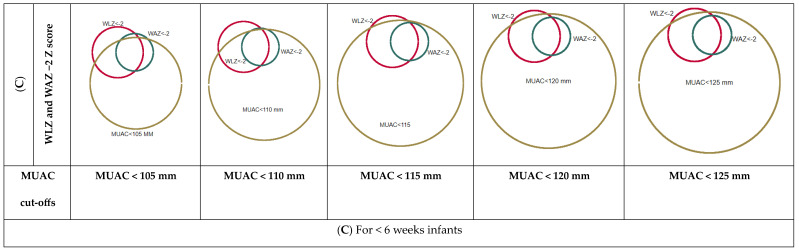
A proportional Venn diagram that visualises malnutrition caseloads in infants u6m (all infants, those aged between 6 weeks to 6 months and younger infants aged < 6 weeks): it visualises the total malnutrition caseloads of both sample population and national estimates captured by the different MUAC thresholds compared with total cases identified by wasting (WLZ < −2), underweight cases (WAZ < −2), or withered cases that were captured by each specific MUAC threshold ranging from 105 to 125 mm. (**A**) For 0–6 months infants. (**B**) For >= 6 weeks to 6 months infants. (**C**) For < 6 weeks infants.

Figure 3 shows the proportional Venn diagram for infants u6m, indicating the relationship between caseload for wasting and caseload for underweight across the different MUAC cut-off points and Z scores among infants aged < 6 weeks and >= 6 weeks to 6 months. For infants aged 0 to 6 months, malnutrition caseload as defined by MUAC increases as its threshold increases from 105 mm through 125 mm against the caseload, identified by an WLZ and/or WAZ of <−2. An MUAC threshold of 120 mm captures almost all cases identified by either low WAZ and/or WLZ criteria (Figure 3A). Most of the severe malnutrition caseload, defined by both WLZ and/or WAZ being at < −3, might be captured at an MUAC threshold of <110 mm (Figure 3A).

Separate analyses based on the following two different age groups across the different MUAC thresholds shows similar scenarios. Among infants aged between 6 weeks and 6 months, an MUAC threshold of <120mm includes most malnourished infants u6m identified by low WLZ and/or WAZ of <−2 of the WHO criteria (Figure 3B).

Similarly, an MUAC threshold of <115 mm might capture almost all severely malnourished infants aged between 6 weeks and 6 months identified by severely low WLZ and/or WAZ of <−3 of the WHO criteria (Figure 3B). However, for infants aged < 6 weeks, an MUAC cut-off of <110 mm might identify a large number of malnourished cases compared with cases identified by low WAZ (−2) and WLZ (−2) criteria [Figure 3C].

### 3.4. Malnutrition Caseload Categories and Anthropometric Overlap Across the Different MUAC Thresholds

The number of infants at risk of poor growth and development eligible for treatment aged 6 weeks to 6 months, as identified by underweight (WAZ < −2) or wasting (WLZ < −2) or withering (MUAC < 110 mm) in the WHO-2023 criteria, was 180 (19.2%; 95% CI: 16.8, 21.9); this is significantly greater than the caseload identified by the old WLZ < −3 WHO-2013 criteria of 25 (2.4%; 95% CI: 1.6, 3.5) at *p* value < 0.001. 

Moreover, the number of infants at risk of poor growth and development eligible for treatment aged < 6 weeks as identified by wasting (WLZ < −2) or underweight (WAZ < −2) of the WHO-2023 criteria was 14 (15.1%; 95% CI: 9.1, 24), which is also significantly greater than the caseload identified via WLZ < −3 WHO-2013 criteria.

Table 2 summarises malnutrition caseload and anthropometric overlap across three age groups with different MUAC thresholds, both in our study and at the national level. Overall, infants at the national level who are at risk of poor growth and development and are eligible for treatment are estimated to number 320,618 cases for those aged 6 weeks to 6 months and 24,938 cases for infants under 6 weeks, as identified by the WHO-2023 criteria; those cumulative figures exceed the 44,531 severe wasting cases at the national level, which were identified using the previous WLZ < −3 WHO-2013 criteria (Table 2).

Of 133 (12.9%) underweight infants aged 0 to 6 months diagnosed by low WAZ < −2 of WHO-2023 criteria, 122 (91.7%) of the cases were overlapped with withering at an MUAC threshold of 125 mm. This means that, of the current nationally estimated 236,901 underweight infants u6m, 217,308 infants were overlapped with withering at the same MUAC cut-off. However, only 59 (44.4%) out of the total cases diagnosed by low WAZ overlapped with wasting (WLZ < −2) in the WHO-2023 criteria. This means that, out of the nationally estimated underweight infants, fewer than 50%, 105,092 infants, were overlapped with wasting. Additionally, of the 112 (10.9%) wasted cases identified by low WLZ, 91 (81.3%) of them were also overlapped with withering at an MUAC threshold of <125 mm. If extrapolated nationally, of the nationally estimated 199,496 wasted infants, 162,090 of them were overlapped with withering at an MUAC cut-off of <125 mm (Table 2).

Overall, among the total infants included in this analysis, 514 (49.9%; 95% CI: 46.8, 53, at *p* value < 0.001) malnourished infants were diagnosed as withered, or wasted, or underweight at MUAC cut-off of <125 mm (Table 2). If these same percentages applied nationally, out of the nationally estimated 1,834,643 infants u6m, 915,541 of them were found to have any of the three malnutrition forms at this MUAC cut-off (Table 2).

In our subgroup analysis, 115 (91.3%) out of 126 (13.4%) total underweight infants aged 6 weeks to 6 months with low WAZ were also overlapped with withering at a low MUAC threshold of <125 mm. If extrapolated to Ethiopia as a whole, out of the nationally estimated 224,433 underweight infants in this age group, 204,839 of them were overlapped with withering at this same MUAC cut-offs. Plus, 79 (79%) out of 100 (10.7%) total low WLZ cases were also overlapped with withering at the same MUAC threshold. This means that, of 178,121 nationally estimated wasted infants in this age group, 140,716 of them also had withering at the MUAC cut-off of <125 mm (Table 2).

Overall, 423 (45.1%; 95% CI: 42, 48.4 at a *p* value of <0.001) of the total infants included in this age group were classified as having either withering, wasting, or being underweight at the MUAC cut-off < 125 mm.

This means that, of the 1,668,991 nationally estimated infants in this age group, 753,451 of them were found to have any of the three forms of malnutrition (Table 2).

Among infants aged < 6 weeks, 12 (12.9%) of the total wasted cases diagnosed by low WLZ were similarly identified by withering at a low MUAC threshold of <115 mm. This means that all 21,375 nationally estimated wasted infants were overlapped with withering at this same MUAC cut-off. Meanwhile, of the seven (7.5%) total cases diagnosed by low WAZ; all of the cases were also identified by withering at the same low MUAC cut-off. This means that all 12,469 underweight cases estimated nationally were overlapped with withering at this MUAC cut-off (Table 2).

Overall, among all infants included in the analysis, 76 (81.7%; 95% CI: 72.4, 88.4 at a *p* value of <0.001) were identified as wasting, withering, or underweight at MUAC cut-offs of <115 mm. Overall, 135,373 out of the 165,653 nationally estimated infants have one of the three forms of malnutrition (Table 2).

### 3.5. Exposure of Infants u6m for Malnutrition Across the Different Characteristics Using the New WHO-2023 and Old WHO-2013Anthropometric Indicators of Malnutrition

Table 3 summarises exposure for malnutrition among infants u6m using the new WHO-2023 and old WHO-2013 diagnostic criteria. 

Among the total identified infants u6m with different kinds of malnutrition, more male infants had malnutrition than females, having 70 (12.3%) wasted, 86 (14.9%) underweight, and 28 (5.2%) withered. Similarly, of the total exposed infants u6m with various forms of malnutrition, more infants from mothers married young, aged < 19 years had malnutrition than their counterparts, with 98 (11.6%) wasted, 114 (13.4%) underweight, and 54 (6.9%) withered [Table 3].

Among the total infants u6m with various forms of malnutrition, nearly half and greater number of those who were non-exclusively breastfed (NEBF) experienced malnutrition, including 65 (12.3%) with wasting, 77 (14.4%) underweight, and 32 (6.2%) withered. Additionally, many infants from households without access to tap water for drinking had different forms of malnutrition, with 70 (11.8%) wasted, 87 (14.5%) underweight, and 42 (7.7%) withered. Furthermore, a greater number of infants from Deder had various forms of malnutrition compared to those from Jimma, with 67 (15.7%) wasted, 79 (18.1%) underweight, and 27 (6.7%) withered (Table 3). 

Among the total number of severely malnourished infants u6m as defined by the WHO-2013 criteria (WLZ < −3), more than one-third; 17 (4%) of the infants in Deder were found to have severe wasting compared to those from Jimma. Additionally, a greater number of male infants, 17 (3%), were identified as severely wasted versus 11 (2.4%) of female infants u6m (Table 3). 

## 4. Discussion

We found substantial overlaps among anthropometric indicators of malnutrition among infants u6m, as defined by the recently established WHO-2023 criteria for infants u6m at risk of poor growth and development. However, the overlap was not perfect with the three indicators (low MUAC, low WAZ, low WLZ) also identifying different individuals and the extent of overlap varying markedly depending on the threshold used and age group.

Among infants aged >= 6 weeks to 6 months, withering at a high MUAC threshold of <125 mm overlapped with more than 90% of underweight (WAZ < −2) infants. This directly reflected that, of the nationally estimated 224,333 underweight infants u6m in Ethiopia, 204,839 of them were also classified as withered at the same MUAC cut-off. Also, nearly 80% of wasted (WLZ < −2) cases were similarly identified as having withering at this same MUAC cut-off. This directly reflects that, 140,716/178,121 wasted infants in Ethiopia can similarly be diagnosed as withering at an MUAC cut-off of <125 mm.

Moreover, 423 (45.1%) caseloads were identified through withering, wasting, or underweight. If applied nationally, an estimated 753,451 of 1,668,991 infants of this age group in Ethiopia would be classified as at risk of poor growth and development and thus eligible for programme enrolment/support.

This highlights the fact that though there are many common factors and underlying aetiologies, different anthropometric indicators capture different aspects of nutritional (as well as health) status and are not directly synonymous [40,41]. What matters is how well different indicators identify infants most at risk. However, further investigation is necessary to obtain setting-specific evidence and determine the optimal cut-offs for programme enrolment based on each country’s resources and programme capacity [10,40,41].

Unlike other concurrent malnutrition forms found in this study including withering with underweight or withering with wasting, only 44.4% of wasted cases were overlapped with underweight cases at a low MUAC threshold of <125mm. These data reflect that fewer than 50%, 105,092 of 236,901 underweight WAZ < −2) infants u6m in Ethiopia, were overlapped with wasting (WLZ < −2).

Low WAZ may be a preferable indicator to low WLZ as length-based measurement has poor reliability and poor performance in capturing high-risk cases [10,42,43]. Evidence suggests that those at highest risk of death are best targeted by using MUAC and/or WAZ [42]; this could work well in our setting given the marked overlap of withering and underweight in our sample.

We also showed that an MUAC threshold of <110 mm as specified by the WHO-2023 criteria capture only about a third of all potential cases diagnosed by low WAZ (<−2) and/or low WLZ (<−2). Increasing the ‘low MUAC’ cut-offs from < 110 mm to 125 mm unsurprisingly results in more cases identified. Using such higher MUAC thresholds, perhaps in combination with WAZ, might better identify infants at hight risk of death [44,45]. Even for infants aged less than 6 weeks, an MUAC threshold of <115 mm works well to better predict mortality [46]. Therefore, the WHO’s recommendation of an MUAC threshold of <110 mm for infants aged between 6 weeks and 6 months might miss cases who could benefit from support [45,47].

The added benefits of MUAC-based enrolment are that it is simple, practical, and easy to use for both healthcare professionals and even mothers [13,14,15,48]. Its inclusion for this age group of u6m is thus a major move forward, even if there is a scope to refine thresholds in the future. These might be universal or might even be informed by analyses such as ours and tailored to specific countries to optimise both overlap with other indicators and the ability to identify high-risk infants while simultaneously keeping programme caseloads manageable.

Others reasons why MUAC-based programming has future promise for infants aged u6m is its association with low birth weight infant and neonatal mortality [49,50]. The link between low birth weight infants and mortality or slow growth velocity due to intrauterine growth restriction is well established [51,52]. Therefore, MUAC would be considered to identify those younger infants at higher risk of poor growth and development. Another key finding of our study was that the current WHO-2023 criteria for infants u6m at risk of poor growth and development u6m risk identify many more cases than the previous WHO-2013 criteria. This concurs with what others have found [10,42,53] and is unsurprising given the new measures as well as the higher thresholds for programme enrolment.

In proposing alterative MUAC cut-offs to policymakers and programmers, we suggest that less conservative MUAC cut-offs are more inclusive and capture more cases of wasting or underweight or withering as per the new WHO-2023 set criteria [12]. This could be helpful for MUAC-based malnutrition programs focusing on infants u6m in the same way, as there are MUAC-only programmes for older children [9,54]. These are especially valuable where WLZ and WAZ measurements are difficult to obtain [45,47,55].

We also recognise that future changes to anthropometric enrolment criteria should be carefully considered, with nuanced examinations of the associated trade-offs associated with greater/fewer patients’ number, particularly in resource-limited settings. Programmes might require more resources, such as skilled healthcare professionals, medical supplies, and infrastructure comparable with the existing program services [16,17,56]. Therefore, various countries with limited resources should carefully assess their own caseload rollout criteria for future program feasibility and sustainability, as infants u6m deserve a more comprehensive and multidimensional package of care [12,18].

We also found that, among the total identified infants u6m with different forms of malnutrition, greater number of non-exclusively breastfed (NEBF) infants u6m were affected by one of the distinct forms of malnutrition. This is also consistent with the previous literature [57,58]. Though EBF is key to prevent malnutrition among infants u6m, particularly in resource-limited settings, their vulnerability to malnutrition is beyond their exclusivity of breastfeeding [57]. Maternal nutrition, maternal quality of life, breastfeeding efficacy, infant malnutrition at birth [59,60,61,62,63,64,65,66], and poor maternal mental health [67,68,69,70] are significant determinants of malnutrition among infants u6m. Therefore, given many risk factors associated with infant malnutrition, there is a need for multi-pronged solution addressing issues beyond EBF alone.

We also identified that, among the total identified infants u6m with different forms of malnutrition, greater number of infants u6m in our Deder study site had different forms of malnutrition, which is risky for poor growth and development. This might be because Deder’s high number of internally displaced people and repeated droughts contribute to poor maternal quality of life and associated infant malnutrition [26,27,28,62,71]. Lactating mothers require extra nutrients to adequately breastfeed infants u6m [59], and those in vulnerable areas like Deder often face inadequate diets and are at thus at risk of maternal undernutrition, which in turn is linked to malnutrition in infants u6m [60,61,63,64].

### 4.1. Theoretical and Practical Policy Implications

Overall, the introduction of this new WHO-2023 malnutrition management guide-line for infants u6m at risk for poor growth and development is an important opportunity to estimate and project the current national burden of malnutrition in infants u6m and the associated caseload implication for future enrolment and program management.

While more longitudinal data are required to identify the risks linked with infant mortality/morbidity/poor development under the new criteria, our study points to a wider need to consider infant u6m malnutrition and how it is managed. This is because vulnerable infants u6m with different anthropometric deficits might have a variety of underlying clinical and social issues, and these need to be considered in assessment and program management [7,11,72,73,74].

Another issue raised by our data is how infants identified by differing criteria (and by different overlaps) might vary and whether their clinical/management needs are also different. They might, for example, require a more diverse and integrated package of clinical care to lower their heightened risk of mortality/morbidity/poor development [11,12]. If infants with overlapping anthropometric deficits do not receive timely and effective evidence-based clinical care, they are at a higher risk of dying prematurely [40].

Whilst ideal-world situations would enrol all vulnerable infants, real-world programmes often have to prioritise and make difficult trade-offs. A high number of malnutrition caseload identified through expanded anthropometric criteria may exceed the capacity of existing healthcare systems and available resources [16,17,56]. Therefore, country-level readiness in terms of skilled and well-trained manpower and enhanced infrastructure with all of the required equipment is the number one priority [16,17]. Therefore, we are directly calling on policymakers and program managers to give special priority to this current WHO-2023 malnutrition management guideline for its country-specific rollout and maximal implementation without program stretch, particularly in resource-limited settings around the world [19].

### 4.2. Strengths and Limitations

Our study has several strengths. To the best of ours’ knowledge, this is the first study added to the malnutrition literature investigating the potential implications of adopting the new WHO-2023 guideline for severe malnutrition. This is vital and timely, given that many countries are currently in the process of working out how to implement the new guideline at the national level. Our illustration of possible caseload implications using different MUAC thresholds is especially helpful given that some programmes might consider MUAC-only programming and would want to know how many of the infants defined by other anthropometric measures would still be captured (and how many would be missed) by such an approach.

Whilst Ethiopian epidemiology cannot be applied blindly to other settings, our method of exploring both overlap between indicators and the effect of different thresholds on potential caseload numbers can be repeated in other areas. 

This would help researchers, programmers, and policymakers to better understand their own local malnutrition epidemiology before incorporating the new WHO-2023 guideline into national/programme protocols for severe malnutrition management. It is vital to clearly understand possible caseloads before fully endorsing and scaling any programme.

We also acknowledge limitations. Most importantly, our study is only a cross-sectional study; we do not have short or long-term outcome data to know which indicators or which combination of indicators best identify the highest risk infants who should be the focus of treatment [11,75,76].

We also note that some of our variables were self-reported and that other important variables such as prematurity and LBW were not available. Additionally, we do not have systematic data on other criteria specified by the WHO also define an infant who is ‘at risk of poor growth and development’. Notably we lack data on oedema, which is key to defining severe malnutrition. We also did not have data on growth patterns or maternal concern with growth. We did have some data on maternal and infant risk but did not include it in our caseload calculations, as its use is likely to vary markedly in different programmes.

National infant mortality might also reduce the number of infants estimated in our study; however, it is not so high in Ethiopia and is unlikely to significantly affect our overall estimates and key messages. Our projections were directly derived from our data and the recently updated birth rate in Ethiopia [38], ensuring that the caseload information is nationally representative.

We were unable to visually represent the caseload of severe malnutrition among infants aged under six weeks using proportional Venn diagrams due to insufficient data to accurately position the circles. However, our analysis provided clear quantitative information and reporting on these findings. We clearly interpreted the MUAC cut-offs at which the maximal cases can be captured against the low WLZ (−3) and low WAZ (−3) scores without greatly affecting the caseload for program inclusion.

Finally, as well as data on caseload, we did not have data on the practicalities of different measurements. Future research is warranted to assess the acceptability and feasibility of different MUAC-cut-off thresholds and other anthropometric indicators in resource-limited settings. It would also be important to future studies to collect longitudinal data to investigate the sensitivity/specificity of anthropometric indictors in identifying mortality/morbidity/poor development rates of at-risk infants u6m.

## 5. Conclusions

New WHO-2023 criteria markedly increase infant u6m caseloads compared with old WHO-2013 criteria. Whilst there are strong overlaps between low WAZ, low WLZ, and low MUAC, they capture different groups of infants. Future data are needed to know which of the criteria (or combination of criteria) best identify infants at the highest risk of mortality/morbidity/poor development. There might be a case for increasing MUAC thresholds for MUAC-focused programming, where WAZ and/or WLZ measurement is difficult.

## Figures and Tables

**Table 1:** Socio-demographic and economic characteristics of u6m infant–mother pairs (N = 1060).

Variables	Category	Number	%
Infant sex	Male	588	55.5
Infant age in months	<2 months	275	25.9
	2 to <4 months	454	42.8
	>=4 month	331	31.2
Twin category	Singleton	1043	98.4
	Non-singleton	17	1.6
Low birth weight (self-reported)	Yes	10	0.9
Infant age in weeks	<6	103	9.7
	6 to <25	957	90.3
Maternal age	<19 years	94	8.9
	>=19 years	966	91.1
Maternal age at marriage	<19 years	862	81.3
	>=19 years	198	18.7
Maternal education	No education	434	40.9
	Primary	485	45.8
	Secondary	96	9.1
	Tertiary	45	4.2
Maternal mental health (PHQ-9 questionnaire)	Normal	941	89.4
	Mild	80	7.6
	Moderate	0	0.0
	Severe	31	2.9
Infant exclusively breastfed	Yes	519	49.0
Infant’s exact birth date know	Yes	878	82.8
Household head’s education	No education	340	34.1
	Primary	452	45.4
	Secondary	139	14
	Tertiary	65	6.5
Family size	<5	627	59.2
	>=5	433	40.8
Mobile phone for communication	Yes	385	36.3
Grandmother helps with child care	Yes	381	35.9
Drinking water type	Tap water	449	42.4
	Non-tap water	611	57.6
Wealth status (internally generated quintiles)	Poorest	212	20.0
	Poor	213	20.1
	Middle	212	20.0
	Rich	212	20.0
	Richest	211	19.9
Area	Jimma (livelihood-secure)	623	58.8
	Deder (livelihood-insecure)	437	41.2

**Table 2:** Malnutrition caseload and anthropometric overlaps in infants u6m (all infants, those aged between 6 weeks to 6 months and younger infants aged <6 weeks) at different MUAC thresholds both in our study and at national level.

**All Infants in Our Study Aged 0 to 6 Months (n = 1030)**										
**Indicators of Malnutrition**	**MUAC Thresholds with** **WAZ and WLZ < −2 Z Score**					**MUAC Thresholds with** **WAZ and WLZ < −3 Z Score**				
	MUAC < 105 mm	MUAC < 110 mm	MUAC < 115 mm	MUAC < 120 mm	MUAC < 125 mm	MUAC < 105 mm	MUAC < 110 mm	MUAC < 115 mm	MUAC < 120 mm	MUAC < 125 mm
Withering (%)	69 (6.7)	123 (11.9)	196 (19)	326 (31.7)	486 (47.2)	69 (6.7)	123 (11.9)	196 (19)	326 (31.7)	486 (47.2)
Wasting (%)	112 (10.9)	112 (10.9)	112 (10.9)	112 (10.9)	112 (10.9)	25 (2.4)	25 (2.4)	25 (2.4)	25 (2.4)	25 (2.4)
Underweight (%)	133 (12.9)	133 (12.9)	133 (12.9)	133 (12.9)	133 (12.9)	47 (4.6)	47 (4.6)	47 (4.6)	47 (4.6)	47 (4.6)
Wasting and withering (%)	19 (1.8)	31 (3)	52 (5)	81 (7.9)	91 (8.8)	6 (0.6)	10 (1)	10 (1)	18 (1.7)	21 (2)
Underweight and withering (%)	32 (3.1)	59 (5.7)	82 (8)	112 (10.9)	122 (11.8)	21 (2)	28 (2.7)	33 (3.2)	39 (3.8)	43 (4.2)
Wasting and underweight (%)	59 (5.7)	59 (5.7)	59 (5.7)	59 (5.7)	59 (5.7)	12 (1.2)	12 (1.2)	12 (1.2)	12 (1.2)	12 (1.2)
Wasting and underweight and withering (%)	14 (1.4)	25 (2.4)	39 (3.8)	53 (5.1)	55 (5.3)	6 (0.6)	8 (0.8)	8 (0.8)	8 (0.8)	10 (1)
Wasting or underweight or withering (%)	218 (21.2)	244 (23.7)	287 (27.9)	372 (36.1)	514 (49.9)	108 (10.5)	153 (14.9)	221 (21.5)	337 (32.7)	492 (47.8)
**All infants aged 0 to 6 months based on national estimates (n = 1,834,643)**										
Withering (%)	122,904 (6.7)	219,089 (11.9)	349,117 (19.3)	580,674 (31.7)	865,667 (47.2)	122,904 (6.7)	219,089 (11.9)	349,117 (19.3)	580,674 (31.7)	865,667 (47.2)
Wasting (%)	199,496 (10.9)	199,496 (10.9)	199,496 (10.9)	199,496 (10.9)	199,496 (10.9)	44,531 (2.4)	44,531 (2.4)	44,531 (2.4)	44,531 (2.4)	44,531 (2.4)
Underweight (%)	236,901 (12.9)	236,901 (12.9)	236,901 (12.9)	236,901 (12.9)	236,901 (12.9)	83,717 (4.6)	83,717 (4.6)	83,717 (4.6)	83,717 (4.6)	83,717 (4.6)
Wasting and withering (%)	33,843 (1.8)	55,218 (3)	92,623 (5)	144,278 (7.9)	162,090 (8.8)	10,688 (0.6)	17,813 (1)	17,813 (1)	32,062 (1.7)	37,406 (2)
Underweight and withering (%)	56,999 (3.1)	105,092 (5.7)	146, 059 (8)	199,496 (10.9)	217,308 (11.8)	37,406 (2)	49,874 (2.7)	58,780 (3.2)	69,468 (3.8)	76,592 (4.2)

**Cont…**	**MUAC Thresholds with** **WAZ and WLZ < −2 Z Score**					**MUAC Thresholds with** **WAZ and WLZ < −3 Z Score**				
	MUAC < 105 mm	MUAC < 110 mm	MUAC < 115 mm	MUAC < 120 mm	MUAC < 125 mm	MUAC < 105 mm	MUAC < 110 mm	MUAC < 115 mm	MUAC < 120 mm	MUAC < 125 mm
**All infants aged 0 to 6 months based on national estimates (n = 1,834,643)—continued**										
Wasting and underweight (%)	105,092 (5.7)	105,092 (5.7)	105,092 (5.7)	105,092 (5.7)	105,092 (5.7)	21,375 (1.2)	21,375 (1.2)	21,375 (1.2)	21,375 (1.2)	21,375 (1.2)
Wasting and underweight and withering (%)	24,937 (1.4)	44,531 (2.4)	69,468 (3.8)	94,404 (5.1)	97,967 (5.3)	10,688 (0.6)	14,250 (0.8)	14,250 (0.8)	14,250 (0.8)	17,813 (1.0)
Wasting or underweight or withering (%)	388,304 (21.2)	434,615 (23.7)	511,207 (27.9)	662,609 (36.1)	915,541 (49.9)	192,371 (10.5)	272,525 (14.9)	393,647 (21.5)	600,267 (32.7)	876,354 (47.8)
**Infants aged >= 6 weeks to 6 months in our study (n = 937)**										
Withering (%)	28 (3)	62 (6.6)	120 (12.8)	239 (25.5)	395 (42.2)	28 (3)	62 (6.6)	120 (12.8)	239 (25.5)	395 (42.2)
Wasting (%)	100 (10.7)	100 (10.7)	100 (10.7)	100 (10.7)	100 (10.7)	25 (2.7)	25 (2.7)	25 (2.7)	25 (2.7)	25 (2.7)
Underweight (%)	126 (13.4)	126 (13.4)	126 (13.4)	126 (13.4)	126 (13.4)	44 (4.7)	44 (4.7)	44 (4.7)	44 (4.7)	44 (4.7)
Wasting and withering (%)	11 (1.2)	22 (2.3)	40 (4.3)	69 (7.4)	79 (8.4)	6 (0.6)	10 (1.1)	10 (1.1)	18 (1.9)	21 (2.2)
Underweight and withering (%)	27 (2.9)	53 (5.7)	75 (8)	105 (11.2)	115 (12.3)	18 (1.9)	25 (2.7)	30 (3.2)	36 (3.8)	40 (4.3)
Wasting and underweight (%)	54 (5.8)	54 (5.8)	54 (5.8)	54 (5.8)	54 (5.8)	12 (1.3)	12 (1.3)	12 (1.3)	12 (1.3)	12 (1.3)
Wasting and underweight and withering (%)	11 (1.2)	21 (2.2)	34 (3.6)	48 (5.1)	50 (5.3)	6 (0.6)	8 (0.9)	8 (0.9)	8 (0.9)	10 (1.1)
Wasting or underweight or withering (%)	173 (18.5)	180 (19.2)	211 (22.5)	285 (30.4)	423 (45.1)	67 (7.2)	92 (9.8)	145 (15.5)	250 (26.7)	401 (42.8)
**Infants aged >= 6 weeks to 6 months based on national estimates (n = 1,668,991)**										
Withering (%)	49,874 (3)	110,435 (6.6)	213,745 (12.8)	425,709 (25.5)	703,577 (42.2)	49,874 (3)	110,435 (6.6)	213,745 (12.8)	425,709 (25.5)	703,577 (42.2)
Wasting (%)	178,121 (10.7)	178,121 (10.7)	178,121 (10.7)	178,121 (10.7)	178,121 (10.7)	44,531 (2.7)	44,531 (2.7)	44,531 (2.7)	44,531 (2.7)	44,531 (2.7)
Underweight (%)	224,433 (13.4)	224,433 (13.4)	224,433 (13.4)	224,433 (13.4)	224,433 (13.4)	78,374 (4.7)	78,374 (4.7)	78,374 (4.7)	78,374 (4.7)	78,374 (4.7)
Wasting and withering (%)	19,594 (1.2)	39,187 (2.3)	71,249 (4.3)	122,904 (7.4)	140,716 (8.4)	10,688 (0.6)	17,813 (1.1)	17,813 (1.1)	32,062 (1.9)	37,406 (2.2)
Underweight and withering (%)	48,093 (2.9)	94,404 (5.7)	133, 591 (8)	187,027 (11.2)	204,839 (12.3)	32,062 (1.9)	44,531 (2.7)	53,437 (3.2)	64,124 (3.8)	71,249 (4.3)
Wasting and underweight (%)	96,186 (5.8)	96,186 (5.8)	96,186 (5.8)	96,186 (5.8)	96,186 (5.8)	21,375 (1.3)	21,375 (1.3)	21,375 (1.3)	21,375 (1.3)	21,375 (1.3)
Wasting and underweight and withering (%)	19,594 (1.2)	37,406 (2.2)	60,562 (3.6)	85,498 (5.1)	89,061 (5.3)	10,688 (0.6)	14,250 (0.9)	14,250 (0.9)	14,250 (0.9)	17,813 (1.1)
Wasting or underweight or withering (%)	308,149 (18.5)	320,618 (19.2)	375,835 (22.5)	507,645 (30.4)	753,451 (45.1)	119,341 (7.2)	163,872 (9.8)	258,276 (15.5)	445,302 (26.7)	714,265 (42.8)
**Cont…**	**MUAC Thresholds with** **WAZ and WLZ < −2 Z Score**					**MUAC Thresholds with** **WAZ and WLZ < −3 Z Score**				
	MUAC <105 mm	MUAC < 110 mm	MUAC < 115 mm	MUAC < 120 mm	MUAC < 125 mm	MUAC < 105 mm	MUAC < 110 mm	MUAC < 115 mm	MUAC < 120 mm	MUAC < 125 mm
**Infants aged < 6 weeks in our study (n = 93)**										
Withering (%)	41 (44.1)	61 (65.6)	76 (81.7)	87 (93.5)	91 (97.8)	41 (44.1)	61 (65.6)	76 (81.7)	87 (93.5)	91 (97.8)
Wasting (%)	12 (12.9)	12 (12.9)	12 (12.9)	12 (12.9)	12 (12.9)	-	-	-	-	-
Underweight (%)	7 (7.5)	7 (7.5)	7 (7.5)	7 (7.5)	7 (7.5)	3 (3.2)	3 (3.2)	3 (3.2)	3 (3.2)	3 (3.2)
Wasting and withering (%)	8 (8.6)	9 (10)	12 (12.9)	12 (12.9)	12 (12.9)	-	-	-	-	-
Underweight and withering (%)	5 (5.4)	6 (6.5)	7 (7.5)	7 (7.5)	7 (7.5)	3 (3.2)	3 (3.2)	3 (3.2)	3 (3.2)	3 (3.2)
Wasting and underweight (%)	5 (5.4)	5 (5.4)	5 (5.4)	5 (5.4)	5 (5.4)	-	-	-	-	-
Wasting and underweight& withering(%)	3 (3.2)	4 (4.3)	5 (5.4)	5 (5.4)	5 (5.4)	-	-	-	-	-
Wasting or underweight or withering (%)	45 (48.3)	64 (68.8)	76 (81.7)	87 (93.5)	91 (97.8)	41 (44.1)	61 (65.6)	76 (81.7)	87 (93.5)	91 (97.8)
**Infants aged < 6 weeks based on national estimates (n= 165,653)**										
Withering (%)	73,030 (44.1)	108,655 (65.6)	135,373 (81.7)	154,966 (93.5)	162,091 (97.8)	73,030 (44.1)	108,655 (65.6)	135,373 (81.7)	154,966 (93.5)	162,091 (97.8)
Wasting (%)	21,375 (12.9)	21,375 (12.9)	21,375 (12.9)	21,375 (12.9)	21,375 (12.9)	-	-	-	-	-
Underweight (%)	12,469 (7.5)	12,469 (7.5)	12,469 (7.5)	12,469 (7.5)	12,469 (7.5)	5344 (3.2)	5344 (3.2)	5344 (3.2)	5344 (3.2)	5344 (3.2)
Wasting and withering (%)	14,250 (8.6)	16,031 (10)	21,375 (12.9)	21,375 (12.9)	21,375 (12.9)	-	-	-	-	-
Underweight and withering (%)	8907 (5.4)	10,688 (6.5)	12,469 (7.5)	17, 761 (10.8)	17, 761 (10.8)	5344 (3.2)	5344 (3.2)	5344 (3.2)	5344 (3.2)	5344 (3.2)
Wasting and underweight (%)	8907 (5.4)	8907 (5.4)	8907 (5.4)	8907 (5.4)	8907 (5.4)	-	-	-	-	-
Wasting and underweight and withering (%)	5344 (3.2)	7125 (4.3)	8907 (5.4)	8907 (5.4)	8907 (5.4)	-	-	-	-	-
Wasting or underweight or withering (%)	80,155 (48.3)	113,998 (68.8)	135,373 (81.7)	154,966 (93.5)	162,091 (97.8)	73,030 (44.1)	108,655 (65.6)	131,810 (81.7)	154, 966 (93.5)	162,091 (97.8)

Footnotes: All infants u6m were estimated using the following formula: (crude fertility rate × total population)/2:(0.029 × 126,527,060)/2 ≈ 1,834,643 infants u6m projected in 2023 Ethiopian infants u6m. Meanwhile, national estimates of infants aged between 6 weeks to 6 months were estimated as: (infants aged >= 6 weeks to 6 months in our sample population/infants aged 0–6 months) × total infants u6m estimated from the national population = (937/1030) × 1,834,643 ≈ 1,668,991 infants aged >= 6 weeks and < 6 months. Additionally, national estimates of infants aged < 6 weeks were also calculated as: (infants aged < 6 weeks in our sample population/infants aged 0–6 months) × total infants u6m estimated from the national population = (93/1030) × 1,834,643 ≈ 165,653 infants aged < 6 weeks. Source: the United Nations Population Fund (UNFPA) population data portal reported in 2023 [38], available at https://pdp.unfpa.org (accessed on 26 November 2024). Wasted, underweight, or withered cases were calculated based on the following formula for probability of mutually unexclusive sets [39]: cases (wasted U underweight U withered) = cases (wasted) + cases (underweight) + cases (withered) − cases (wasted ∩ underweight) − cases (underweight ∩ withered) − cases (wasted ∩ withered) + cases (wasted ∩ underweight ∩ withered).

**Table 3:** Magnitude of malnutrition among infants u6m using the new WHO-2023 and old WHO-2013 diagnostic criteria.

**Variables**	**Characteristics**	**Underweight (WAZ < −2 Z Score of the WHO-2023 Criteria)**	**Withering (MUAC < 11 cm of the WHO-2023 Criteria, Excluding < 6 Weeks Infants)**	**Wasting (WLZ < −2 Z Score of the WHO-2023 Criteria)**	**Severe Wasting Using the WHO-2013 Criteria (WLZ < −3 Z Score, Including < 6 Weeks Infants)**
		**Number (%)**	**Number (%)**	**Number (%)**	**Number (%)**
Infant sex	Male	86/578 (14.9)	28/536 (5.2)	70/571 (12.3)	17/571 (3)
	Female	59/469 (12.6)	40/421 (9.5)	45/462 (9.7)	11/462 (2.4)
Infant age in months	<2 months	35/234 (15)	24/172 (14)	28/262 (10.7)	5/262 (2.7)
	2 to <4 months	51/449 (11.4)	28 /454 (6.2)	44/447 (9.8)	14/447 (3.1)
	>=4 month	59/329 (17.9)	16 /331 (4.8)	43/324 (13.3)	9/324 (2.8)
Twin category	Singleton	134/1030 (13)	63 /942 (6.7)	111/1018 (10.9)	27/1018 (2.7)
	Non-singleton	11/17 (64.7)	5 /15 (33.3)	4/15 (26.7)	1/15 (6.7)
Low birth weight (self-reported)	No	-	-	2/39 (5.1)	-
	Yes	8/10 (80)	-	4/7 (57.1)	-
**Variables** **(continued…)**	**Characteristics**	**Underweight (WAZ < −2 Z Score of the WHO-2023 Criteria)**	**Withering (MUAC < 11 cm of the WHO-2023 Criteria, Excluding < 6 Weeks Infants)**	**Wasting (WLZ < −2 Z Score of the WHO-2023 Criteria)**	**Severe Wasting Using the WHO-2013 Criteria (WLZ < −3 Z Score, Including < 6 Weeks Infants)**
		**Number (%)**	**Number (%)**	**Number (%)**	**Number (%)**
Maternal age	<19 years	12/92 (13)	5 /78 (6.4)	7/91 (7.7)	3/91 (3.3)
	>=19 years	133/955 (13.9)	63 /879 (7.2)	108/942 (11.5)	25/942 (2.7)
Maternal age at marriage	<19 years	114/852 (13.4)	54/779 (6.9)	98/843 (11.6)	24/843 (2.8)
	>=19 years	31/195 (15.9)	14 /178 (7.9)	17/190 (8.9)	4/190 (2.1)
Maternal education	No education	82/429 (19.1)	38 /390 (9.7)	56/422 (13.3)	15/422 (3.6)
	Primary	53/478 (11.1)	26 /444 (5.9)	49/474 (10.3)	11/474 (2.3)
	Secondary	6/95 (6.3)	2 /82 (2.4)	7/94 (7.4)	2/94 (2.1)
	Tertiary	4/45 (8.9)	2 /41 (4.9)	3/43 (7)	-
Maternal mental health (PHQ_9 questionnaire)	Normal	133/929 (14.3)	63/861 (7.3)	94/915 (10.3)	23/915 (2.5)
	Mild	8/79 (10.1)	3/65 (4.6)	10/79 (12.7)	3/79 (3.8)
	Moderate	-	-	-	-
	Severe	2/31 (6.5)	1/24 (4.2)	10/31 (32.3)	2/31 (6.5)
Infant exclusively breastfed	Yes	68/512 (13.3)	36/437 (8.2)	50/504 (9.9)	11/504 (2.2)
	No	77/535 (14.4)	32/520 (6.2)	65/529 (12.3)	17/529 (3.2)
Infant’s exact birth date known	Yes	107/867 (12.3)	48 /781 (6.1)	85/857 (9.9)	23/857 (2.7)
	No	38/180 (21.1)	20 /176 (11.4)	30/176 (17)	5/176 (2.8)
Household head education	No education	58/337 (17.2)	26 /305 (8.5)	42/332 (12.7)	10/332 (3)
	Primary	60/450 (13.3)	25 /409 (6.1)	46/443 (10.4)	13/443 (2.9)
	Secondary	13/138 (9.4)	5 /125 (4)	16/135 (11.9)	2/135 (1.5)
	Tertiary	3/65 (4.6)	2 /58 (3.4)	6/65 (9.2)	1/65 (1.5)
Family size	<5 family	71/620 (11.5)	31 /567 (5.5)	51/612 (8.3)	9/612 (1.5)
	>=5 family	74/427 (17.3)	37/390 (9.5)	64/421 (15.2)	19/421 (4.5)
Mobile phone for communication	Yes	32/380 (8.4)	17/343 (5)	36/375 (9.6)	9/375 (2.4)
	No	113/667 (16.9)	51/614 (8.3)	79/658 (12)	19/658 (2.9)
Grandmother helps with child care	Yes	58/373 (15.5)	27/341 (7.9)	50/367 (13.6)	16/367 (4.4)
	No	87/674 (12.9)	41/616 (6.7)	65/666 (9.8)	12/666 (1.8)
Drinking water type	Tap water	58/448 (12.9)	26 /408 (6.4)	45/438 (10.3)	10/438 (2.3)
	Non-tap water	87/599 (14.5)	42/549 (7.7)	70/595 (11.8)	18/595 (3)
Wealth status (internally generated quintiles)	Poorest	26/209 (12.4)	15 /198 (7.6)	20/208 (9.6)	4/208 (1.9)
	Poor	30/213 (14.1)	13 /197 (6.6)	24/209 (11.5)	10/209 (4.8)
	Middle	31/209 (14.8)	15 /183 (8.2)	23/206 (11.2)	6/206 (2.9)
	Rich	31/210 (14.8)	14 /191 (7.3)	21/205 (10.2)	-
	Richest	27/206 (13.1)	11 /188 (5.9)	27/205 (13.2)	8/205 (3.9)
Area	Jimma (livelihood-secure)	66/610 (10.8)	41 /554 (7.4)	48/607 (7.9)	11/607 (1.8)
	Deder (livelihood-insecure)	79/426 (18.5)	27/403 (6.7)	67/426 (15.7)	17/426 (4)

Footnotes: In Table 3, we presented row percentages of malnourished infants u6m calculated from a different total in each row of a complete case analysis performed using cross-tabulation. In this table, we computed the % exposure of infants u6m to different forms of malnutrition (underweight, wasting, and withering) based on various characteristics that have different samples in each row within a specific variable. For instance, percentage of underweight infants within each characteristic and similar other outcomes’ percentage calculations were made based on the following general formula: (number of underweight infants u6m of that characteristic within that specific variable)/(number of underweight infants u6m + number of non-underweight infants u6m within the same characteristic of that specific variable) × 100.

## Data Availability

The data presented in this study are available upon request from the senior author (the privacy and confidentiality of the data collected from study participants are protected).
